# Prevalence, predictors and economic burden of morbidities among waste-pickers of Mumbai, India: a cross-sectional study

**DOI:** 10.1186/s12995-017-0176-3

**Published:** 2017-10-10

**Authors:** Praveen Chokhandre, Shrikant Singh, Gyan Chandra Kashyap

**Affiliations:** 1 International Institute for Population Sciences, Govandi Station Road Donor, Mumbai, 400088 India; 2 Department of Mathematical Demography & Statistics, International Institute for Population Sciences, Govandi Station Road Deonar, Mumbai, 400088 India

**Keywords:** Injuries, Occupational morbidities, Respiratory illness, Stomach problems, Waste-pickers

## Abstract

**Background:**

The occupation of waste-picking characterised as 3Ds – dangerous, drudgery and demanding. In this context, the study aimed to assess occupational morbidities among the waste-pickers and attempts to identify potential individual level risk factors enhancing health risks. Additionally, economic burden of morbidities has been assessed.

**Methods:**

The burden of the morbidities was assessed and compared with a comparison group through a cross-sectional survey. Waste-pickers (*n* = 200) and a comparison group (*n* = 103) working for at least a year were randomly selected from the communities living on the edge of the Deonar dumping site. The difference in the prevalence of morbidities was tested using the chi-square test. The effect of waste picking resulting the development of morbidities was assessed using the propensity score matching (PSM) method. A multivariate logistic regression model was employed to identify the individual risk factors. T-test has been employed in order to analyse the difference in health care expenditure between waste pickers and non-waste pickers.

**Results:**

The prevalence of morbidities was significantly higher among the waste-pickers, particularly for injuries (75%), respiratory illness (28%), eye infection (29%), and stomach problems (32%), compared to the comparison group (17%, 15%, 18%, and 19% respectively). The results of the PSM method highlighted that waste-picking raised the risk of morbidity for injuries (62%) and respiratory illness (13%). Results of logistic regression suggest that low level of hygiene practices [household cleanliness (OR = 3.23, *p* < 0.00), non-use of soap before meals (OR = 2.65, *p* < 0.05)] and use of recyclable items as a cooking fuel (OR = 2.12, *p* < 0.03) enhanced health risks among the waste pickers when adjusted for the age, duration of work, duration of stay in community and substance use. Additionally, the high prevalence of morbidities among waste pickers resulted into higher healthcare expenditure. Findings of the study suggest that not only healthcare expenditure but persistence of illness and work days lost due to injury/illness is significantly higher among waste pickers compared to non-waste pickers.

**Conclusions:**

The study concluded that waste-picking raised the risk of morbidities as also expenditure on healthcare. Results from the study recommend several measures to lessen the morbidities and thereby incurred healthcare expenditure.

## Background

In the absence of any urban market-based skills, investment and social capital, which are precursors to get gainful employment, many of the migrant urban poor are forced to engage in the filthy occupation of waste picking. On the other hand, the very structure of solid waste management in the cities of developing countries offers an opportunity of survival to millions of waste-pickers [1]. In the developing countries, the rough estimate of waste-pickers was 15 million, of which around 1.5 million belonged to India [2].

The reasons for engaging in the occupation of waste-picking could range from high unemployment to a proliferating amount of solid waste and a growing global market for recycled materials. Waste-pickers work informally and earn a meagre income by collecting and selling recyclable items out of municipal solid waste. While providing the opportunity for survival, waste-picking also has health hazards. Characterised by 3Ds – dangerous, drudgery and demanding – waste-picking leads to fatal and non-fatal morbidities. Past studies indicate that relationship exists between solid waste handling and increased health risks [3–5]. On the other hand, non-fatal morbidities are mainly musculoskeletal in nature. Past studies have revealed a significant relationship between work environment and complaints of musculoskeletal disorders. Workplace activities, such as heavy lifting, manual handling, prolonged bending and repetitive tasks, increase musculoskeletal disorders (MSDs) significantly [6–8]. A study based on waste-pickers suggest that MSDs were significantly higher for the lower back, knees, upper back, shoulders and ankles among the waste-pickers than in the comparison group [9]. In addition to the occupational health risks, their deplorable living conditions, poor hygiene practices and substance use enhance susceptibility to health risks.

In the light of the growing number of the urban poor opting for waste picking and given the nature of waste picking and the associated morbidities, limited studies have been carried out in India. Most of these studies having been done using pilot based data or non-representative samples. Further, these studies have also not taken into account the occupational and individual characteristics which too determine the health risks facing the waste pickers. Moreover, past studies have focused on morbidity prevalence among the waste pickers but have not considered the relative health risks of waste picking compared with the other occupational groups. Following to health risk, to date, hardly any study investigated the health expenditure among waste pickers. Considering this, the present study investigates the occupational health risks among the waste pickers and an attempt has been made to identify the potential risk factors which enhance their health vulnerabilities followed by economic burden of the morbidities.

## Materials and methods

### Design/setting

A cross-sectional study with a comparison group was conducted upon waste-pickers working at the Deonar dumping site – one of the oldest dumping sites in Asia, located in Mumbai.

### Study population

Waste-pickers engaged in waste-picking at the dumping site at least for a year and aged 18 years and above were considered as cases for the study. A group of workers with similar characteristics and engaged in occupations other than waste-picking were considered as the comparison group. They were drawn randomly from in and around the communities where the waste-pickers reside. They were mostly engaged as daily wage labourers, or in embroidery (*zari)* work and other manual occupations.

### Sampling design

There are many slum communities living on the edge of the *Deonar* dumping site. Many of the waste-pickers stay in these communities. Three out of those communities were selected, using probability proportional to size (PPS) sampling. In order to ensure the effective representation of the communities, they were divided into a number of clusters having a household (HH) size ranging from a minimum of 40 to a maximum of 100 HHs. Cluster areas were identified on the basis of natural divisions of the communities. Among the total clusters, approximately 10% were selected using the PPS sampling procedure. In the next stage, mapping and listing operation was carried out in the sampled clusters, where a screener (that is age, occupation and years of working) was canvassed to find targeted participants. Through mapping and listing, we got a sampling frame for the waste-picker households and for the comparison group. Based on the sampling frame, the required number of households were selected from both the groups by using systematic random sampling.

### Sample size

The total sample size for this study was determined based on the proportion of waste-pickers in the selected communities. A community based organisation reported that around 30% of the households in the study area had at least one person engaged in waste-picking. The estimated sample size was 426 households with a *p* value 0.30, a response rate of 0.90 with design effect of 1.20. In order to conduct case-comparison study, the total sample was divided into two equal parts. Finally, a sample of 200 waste-pickers and 200 from comparison group were interviewed (94% response rate). Further, 93 cases were dropped from the comparison group as they were housewives. The results are presented for waste pickers (*n* = 200) and comparison group (*n* = 103). The data was collected during March to July 2014.

### Variables used

#### Response variables

The considered morbidities were based on the past studies and enquired from both the groups. The symptoms of self-reported morbidities of respiratory illness, stomach problem, eye and skin infections, and injuries while picking waste, were recorded for the study. In addition to this, other morbidities that may arise due to the waste picking occupation or due to their poor housing and living conditions were also covered including stomach problem, skin infection, typhoid, jaundice, fever, and cold and cough.

#### Confounding factors

For the present study, age of the respondent, years of working in the occupation, weekly working hours and duration of stay in the community were considered confounding variables, whereas hygiene practices and substance use were considered as effect modifiers.

#### Data analysis

The data was entered in the CSPro.06 software and analysed using the STATA (Version 13.1). Descriptive statistics, such as means, percentages and 95% confidence intervals, were used to describe socio-demographic information. Differences in the prevalence of morbidities among the groups were tested using the chi-square test. Similarly, the difference in healthcare expenditure between waste-pickers and non-waste pickers has been tested using t-test.

## Methods

In order to examine the impact of the waste-picking occupation on the development of the selected morbidities, the study adopted the nearest neighbourhood method of propensity score matching PSM [10, 11]. This approach gives an opportunity to assess the impact of exposure on outcomes through cross-sectional survey data [12–14]. The propensity score is estimated by logistic regression, with the dichotomous exposure/treatment variable. For instance, 1 = exposed to waste-picking occupation; 0 = unexposed to waste-picking occupation, using the associated observed demographic and occupational characteristics of the waste-pickers used as predictor variables.

In this case, the difference in the reported symptoms of the selected morbidities between the waste-pickers and the comparison group can be directly compared to show the impact of the waste-picking occupation on the waste-pickers. This is known as average exposure effect on exposed (AEEE). In order to calculate the impact of waste-picking on the occurrence of the selected morbidities, the average effects in both the groups were weighted by the proportion of the respondents in the exposed and the comparison groups, which measured the increase/decrease in the morbidities due to the waste-picking occupation. Similarly, in order to understand the individual risk factors affecting the health of the waste-pickers for selected morbidities, logistic regression analysis was employed, with adjustment of confounding factors such as age, years of working in the occupation and duration of stay in the community.

## Results

### Demographic and occupational profile

Table 1 suggests that the waste-pickers were comparable with the comparison group in terms of demographic and occupational characteristics. The average age of the waste-pickers was 34 years, similar to 36 years in case of the comparison group, with a standard deviation of 10 years each. A similar pattern was observed with regard to years of working. Marginal difference has been observed in case of average duration of stay in the community between the groups.

**Table 1:** Profile of study groups

Characteristic	Waste-pickers (*n* = 200)	Comparison group (*n* = 103)
Age of workers		
Below 35 years	53.0	47.6
35 & above	47.0	52.4
Mean ± SD	34.0 ± 10.2	35.5 ± 10.3
Duration of stay in the community		
Below 15 years	29.5	43.7
15 & above	70.5	56.3
Mean ± SD	18.7 ± 9.7	15.2 ± 8.8
Working years		
Below 10 yrs.	41.5	37.9
10 & above	58.5	62.1
Mean ± SD	11.2 ± 6.7	11.7 ± 7.4

### Prevalence of morbidities

Table 2 exhibits the prevalence of specific morbidities (during the previous six months) among the waste-pickers and the comparison group. At 75%, the prevalence of injuries was strikingly higher among the waste-pickers; whereas for the comparison group it was 17%. The injuries were mostly lacerations caused by needles or shards of glass, followed by muscle sprain. Similarly, the prevalence of respiratory symptoms was higher among the waste-pickers (28%) than the comparison group (15%). The prevalence of dyspnea and chronic cough particularly was found to be higher among the waste-pickers. Similarly, while considering eye infections, such as redness of eyes, watering of eyes or itching of eyes, the prevalence was higher among the waste-pickers (29%) than the comparison group (18%). For instance, the prevalence of eye soreness (20%) and watering of eyes (13%) was higher among the waste-pickers than the comparison group (19% & 9% respectively). Stomach problems, viz. nausea, gastroenteritis, dysentery, constipation or intestinal pain, were higher among the waste-pickers (32%) than the comparison group (19%). Episodes of gastroenteritis (20%), nausea (14%) and loose motions (14%) especially were found to be higher among the waste pickers than in the comparison group (13%, 2% and 10% respectively).

**Table 2:** Prevalence of morbidities among the waste pickers and the comparison group in the previous six months

	Waste pickers (n = 200)	Comparison group (n = 103)	chi2 (*p*-value)
Injury/Accident	75.0 [0.68 to 0.81]	16.5 [0.09 to 0.23]	(χ2 = 94.03; *p* = 0.000)
Fracture/contusion	3.5	2.9	(χ2 = 0.073; *p* = 0.786)
Muscle sprain	12.5	2.9	(χ2 = 7.451; *p* = 0.006)
Laceration (Needles and glass)	70.0	11.7	(χ2 = 92.58; p = 0.000)
Respiratory infection	28.0 [0.21 to 0.34]	14.6 [0.07 to 0.21]	(χ2 = 6.841; *p* = 0.009)
Dust allergy	12.0	2.9	(χ2 = 6.916; p = 0.009)
Dyspnea	14.5	10.7	(χ2 = 0.866; *p* = 0.352)
Episodes of asthma	4.0	1.9	(χ2 = 0.572; *p* = 0.449)
Chronic cough/Running nose	15.0	2.9	(χ2 = 10.235; *p* = 0.001)
Wheeze and breathlessness	7.5	2.9	(χ2 = 2.560; *p* = 0.110)
Eye infection	29.0 [0.22 to 0.35]	17.5 [0.10 to 0.24]	(χ2 = 4.805; *p* = 0.028)
Eye soreness/infection	19.5	12.6	(χ2 = 2.262; *p* = 0.133)
Watering of eyes	18.5	8.7	(χ2 = 5.031; *p* = 0.025)
Itching of eyes	8.0	1.9	(χ2 = 4.465; *p* = 0.035)
Stomach problem	32.0 [0.25 to 0.38]	19.4 [0.11 to 0.27]	(χ2 = 5.371; *p* = 0.020)
Nausea	14.0	1.9	(χ2 = 11.081; p = 0.001)
Loose motion	14.0	9.7	(χ2 = 1.141; *p* = 0.285)
Gastroenteritis	20.0	12.6	(χ2 = 2.564; *p* = 0.109)
Constipation	7.5	3.8	(χ2 = 1.512; *p* = 0.219)
Skin infection	6.0 [0.02 to 0.09]	2.9 [0.00 to 0.06]	(χ2 = 1.377; *p* = 0.241)
Typhoid/Jaundice	16.0	8.7	(χ2 = 3.064; *p* = 0.080)
Fever/cold & cough	34.5	34.0	(χ2 = 0.008; *p* = 0.928)

Values in square bracket are at 95% Confidence Interval

### Results of PSM for the selected morbidities

The present study attempt to assess the exposure effect of the waste picking occupation on the development of the selected morbidities by estimating the difference in the outcomes between the exposed group (the waste-pickers) and the matched comparison group. Analysis from Table 3 clearly highlights that episodes of injuries were 57% higher among the waste-pickers when compared with the comparison group. Similarly, self-reported symptoms of respiratory illness were found to be higher among the waste-pickers (13%) than the comparison group.

**Table 3:** Average exposure effect and average exposure effect on exposed for the waste picking occupation on the selected morbidities in the previous 6 months

	Average Exposure Effect (AEE)		Average Exposure Effect on Exposed (AEEE)	
	Coef.	[95% Conf. Interval]	Coef.	[95% Conf. Interval]
Injury/Accident	0.57***	(0.463 to 0.672)	0.62***	(0.511 to 0.729)
Respiratory infection	0.13**	(0.017 to 0.233)	0.13**	(0.007 to 0.243)
Eye infection	0.04	(−0.108 to 0.194)	0.03	(−0.172 to 0.232)
Stomach problem	0.02	(−0.191 to 0.231)	0.02	(−0.265 to 0.305)
Typhoid/Jaundice^a^	0.03	(−0.112 to 0.171)	0.01	(−0.189 to 0.199)
Fever/cold & cough^a^	0.05	(−0.072 to 0.171)	0.13**	(0.003 to 0.257)

^a^Morbidities were considered for the 12 months prior to the survey

*p<0.1, **p<0.05, ***p<0.01

### Individual risk factors for morbidities

While analysing the individual risk factors, the results of the logistic regression analysis suggest that advancing years significantly increases the likelihood of respiratory illness (OR = 1.97, *p* = 0.05). Similarly, those who were using wood and other recyclable items as fuel for cooking were significantly more likely to develop respiratory illness (OR = 2.12, *p* = 0.03) compared to those were using liquefied petroleum gases (LPG). In case of stomach problems, household cleaning few days a week (OR = 3.23, *p* = 0.00) and non-use of soap before meals (OR = 2.65, *p* = 0.05) emerged as significant predictors of increased risk of stomach problems (Table 4).

**Table 4:** Logistic regression analysis of independent risk factors for the selected morbidities

	OR	[95% Conf. Int.]
Respiratory illness		
Age		
Below 35 years®		
35 years and above	1.97*	(0.97–3.97)
Fuel for cooking		
LPG®		
Other^a^	2.12**	(1.06 -4.26)
Stomach problem		
Age		
Below 35 years®		
35 years and above	1.73	(0.87–3.43)
Household cleaning		
Daily®		
Some days in a week	3.23***	(1.68–6.21)
Soap use before meals		
Yes®		
No	2.65*	(0.99–7.10)
Availability of waste-water carrying lines		
Yes®		
No	1.68	(0.87–3.21)

®Reference category; Figures are odds ratios with CI at 95%

*p < 0.1, **p < 0.05, ***p < 0.01

^a^Other includes wood, kerosene and material found at the dumping site and used as fuel for cooking

Model is additionally adjusted for substance use, years of working and years of stay in the communities

### Economic burden of the morbidities

Along with the prevalence of morbidities among the waste-pickers, the paper attempt to assess the economic burden of the morbidities in terms of expenditure incurred on treatment, work days lost and persistence of illness among waste-pickers (Table 5). Findings suggest that the mean expenditure was significantly higher among the waste-pickers (₹1736) than the non-waste pickers (₹993). Similarly, the persistence of illness was significantly higher for the waste-pickers (31 days) than the comparison group (19 days). Further, findings suggest that the mean number of work days lost due to morbidities was significantly higher among the waste-pickers (18 days) than the comparison group (11 days). A significant difference in healthcare expenditure has been observed among the waste-pickers by the type of healthcare facility. For instance, the average expenditure incurred at a government healthcare facility for morbidities/injuries was ₹ 582, whereas the corresponding figure for private health facility was ₹1137.

**Table 5:** Economic Burden of the morbidities among waste-pickers and non-waste pickers

	Mean (95% CI)	t-test
Health care expenditure on injuries/illness (INR Rupees)		
Waste pickers	1736 (1248–2225)	*t* = 1.94; *p* = 0.053
Non-waste pickers	993 (535–1451)	
Persistence of injury/illness (days)		
Waste pickers	31 (19–42)	*t* = 1.41; *p* = 0.078
Non-waste pickers	19 (13–25)	
Total work days lost due to injury/illness (days)		
Waste pickers	18 (15–21)	*t* = 2.80; *p* = 0.005
Non-waste pickers	11 (7–14)	
Total health care expenditure among waste pickers by type of facility (INR Rupees)		
Government	582 (364–800)	*t* = 2.05; *p* = 0.041
Private	1137 (670–1602)	

Injuries/illness comprise injuries, respiratory illness, stomach problem, eye infection, skin infection, typhoid/jaundice and fever/cold/cough

## Discussion

The present study examine the prevalence of morbidities among waste-pickers by comparing with a comparison group. Results suggest that the prevalence of injuries was strikingly higher among the waste-pickers (75%) compared to the comparison group (17%). The injuries were mostly due to lacerations caused by shards of glass, followed by muscle sprain. Field insights revealed that there were frequent incidences of serious injuries and deaths occurred when waste-pickers hit by a vehicle or dozer while rushing to collect waste at the time of unloading by waste carrier vehicle. Past studies suggest there is increased risk of hepatitis B and C virus infection due to exposure to sharp instruments during waste collection [15, 16]. Similarly, exposure to fumes at the disposal sites resulted in respiratory problems. The prevalence of respiratory symptoms was found to be significantly higher among the waste-pickers (28%) compared to the comparison group (15%). Particularly, the prevalence of dyspnea (difficulty in breathing) and chronic cough were found to be higher among the waste-pickers. Field insights suggest that the majority of the waste-pickers were not using any protective clothing such as gumboot, gloves and masks, which enhanced their health vulnerabilities. The reason for not using any protective clothing could be their ignorance and poverty [17]. Stomach problems, viz. nausea, dysentery and intestinal pain, were found to be higher among the waste-pickers (32%) than the comparison group (19%). The results of logistic regression clearly highlighted that poor hygiene practices raised the risk of stomach problems. The dumping site being a breeding ground for vectors such as flies, mosquitoes and rats, a large number of diseases and infections are transmitted through these vectors due to their contact with contaminated waste material or water [18]. Results from the propensity score matching method depicted that exposure of waste-picking raised the risk of morbidities such as injuries and respiratory illness when compared to the matched comparison group. Additionally, the nature of their occupation requires the waste-pickers constant bending, which raised the risk of musculoskeletal disorders in many body parts. Waste-pickers have to climb all the way up a pile of garbage to collect waste and climb down with the pile of garbage usually with heavy bags of collected waste rested either on their back, head or shoulders. Hence carrying of heavy weights could also be a reason for the higher prevalence of MSDs among the waste-pickers. Past studies based on waste-pickers exhibit similar results particularly for injuries (82%) [17] respiratory illness (21%) [19] and stomach problems ranging from 39 to 29% [19, 20]. The results from other studies based on waste-pickers did not match with the present study due to dissimilarity in reference period for the reported morbidities.

Additionally, analysis from the study highlighted that the economic burden of the morbidities was significantly higher for the waste-pickers compared to the comparison group. This clearly implies that the economic burden of the morbidities puts them into the poverty trap. Moreover, the health of the waste-pickers is affected at two levels: one, arising out of their poverty and the conditions in which they live; and second, arising out of their exposure to disease and infection due to their occupation. Several authors have extensively recorded the work conditions of the waste-pickers and assessed how they are at the bottom of the socio-economic ladder in terms of income and respect [21–24]. These people live in unhygienic conditions, and the nature of their occupation exposes them to potentially pathogenic bio-aerosols that may lead to the spread of various diseases [25]. The abundance of fleas and offensive odours at the waste disposal sites, along with the lack of proper protective devices, makes them more susceptible to health risk. A clinical study conducted on waste-pickers in Nigeria recorded that they are potential carriers of pathogens that degrade the waste and that they serve as vehicles of transmission of pathogens that are capable of causing diseases in the body [26]. Apart from their occupational health risks, contextual factors, viz. their abysmal living environment, their health and hygiene practices, may further enhance their vulnerability to diseases. Further, several studies have also recorded the higher prevalence of substance use among the waste-pickers [20, 27, 28]. The results of self-reported morbidities could be biased due to subjectivity in responses. Recall bias may also have affected the estimated prevalence of morbidities. Data were collected from waste-pickers who collect the waste from dumping site and not from the other type of waste-pickers who collect waste from road side or community bins and hence generalization of the results must be done with caution.

The work of the waste-pickers is not always appreciated or acknowledged, although they make a positive contribution to the society by reducing the cost of collection and transportation and by reducing the burden of the dumping site. Several studies have tried to estimate the economic contribution of the informal waste sector to the economy as it has a financial impact of several billions of US dollars every year [29]. A more detailed analysis by Medina shows that the work of the waste-pickers makes positive contributions to the society, and with support, these contributions can be even greater. Moreover, waste-picking activity reduces the cost of the proper management of waste and its collection, transportation and proper disposal. Francisco (2009) states that the work of these individuals around the world helps industries by reducing raw material imports [30]. This implies that they contribute to the conservation of natural resources and energy while reducing air and water pollution. They also reduce greenhouse gas emissions through the reuse of materials.

### Suggested strategies to minimize the burden of morbidities among waste-pickers

- This study recommends both preventive and curative measures to minimize the burden of morbidities among waste-pickers.
- Health providers can play a crucial role in reducing the prevalence of morbidities through health education and by increasing the awareness of early signs and symptoms of morbidities.
- Measures should be taken to promote physical exercise as well as the use of protective equipment to reduce work-related musculoskeletal disorders.
- As waste-pickers are unorganized and earn a meagre income, the development of low cost and easy-to-use tools to minimize the occurrence of MSDs would be helpful.
- There is an urgent need of health education particularly related to health and hygiene behaviour among waste-pickers. A study conducted in Thailand exhibited that the significance of the health risk reduction behaviour model (HRRBM) decreased the healthcare costs of individuals and significantly improved knowledge, attitude and practices among the waste-pickers. The percentage of physical symptoms was reduced due to the use of personal protective equipment (PPE) compared to the control group [25].
- Meagre income and a high prevalence of morbidities and healthcare expenditure often leads to poverty [31–33], particularly among the urban poor households [34]. Therefore, it is imperative to promote state sponsored cashless health insurance schemes like Rajiv Gandhi Jeevandayee Arogya Yojna (RGJAY) [35] and Rashtriya Swasthya Bima Yojana (RSBY) [36] among the waste-pickers.

## Conclusions

Several studies including the present study have highlighted that waste-pickers are at a high risk of developing occupational morbidities particularly injuries, respiratory illness, eye infection, stomach problems, typhoid, diarrhoea, and musculoskeletal disorders. Further, individual risk factors such as poor hygiene practices, non-use of protective equipment, inhuman living conditions, and high prevalence of substance use further enhance their health vulnerabilities. This study recommends several possible strategies to abate the episodes of morbidities. Similarly, it propounds the need to promote health insurance among waste-pickers. Overall, in order for them to bargain for their rights in terms of getting a better workplace and recognition of their contribution to solid waste management, collective voices need to be built up.

## Abbreviations

AEE: Average Exposure Effect
AEEE: Average Exposure Effect on Exposed
HHs: Household
HRRBM: Health Risk Reduction Behaviour Model
LPG: Liquefied Petroleum Gases
MSDs: Musculoskeletal Disorder
OR: Odds Ratio
PPE: Personal Protective Equipment
PPS: Probability Proportional to Size
PSM: Propensity Score Matching
RGJAY: Rajiv Gandhi Jeevandayee Arogya Yojna
RSBY: Rashtriya Swasthya Bima Yojana
